# B cells Can Modulate the CD8 Memory T Cell after DNA Vaccination Against Experimental Tuberculosis

**DOI:** 10.1186/1479-0556-9-5

**Published:** 2011-03-14

**Authors:** Luciana P Almeida, Ana PF Trombone, Julio CC Lorenzi, Carolina D Rocha, Thiago Malardo, Isabela C Fontoura, Ana F Gembre, Ricardo LL Silva, Célio L Silva, Ademilson P Castelo, Arlete AM Coelho-Castelo

**Affiliations:** 1 Department of Biochemistry and Immunology, Medical School of Ribeirão Preto, University of São Paulo, Brazil; 2 School of Nursing of Ribeirão Preto, University of São Paulo, Brazil

## Abstract

**Background:**

Although B cells are important as antigen presenting cells (APC) during the immune response, their role in DNA vaccination models is unknown.

**Methods:**

In this study *in vitro *and *in vivo *experiments were performed to evaluate the ability of B cells to protect mice against *Mycobacterium tuberculosis *challenge.

**Results:**

*In vitro *and *in vivo *studies showed that B cells efficiently present antigens after naked plasmid pcDNA3 encoding *M. leprae *65-kDa heat shock protein (pcDNA3-Hsp65) internalization and protect B knock-out (BKO) mice against *Mycobacterium tuberculosis *infection. pcDNA3-Hsp65-transfected B cells adoptively transferred into BKO mice rescued the memory phenotypes and reduced the number of CFU compared to wild-type mice.

**Conclusions:**

These data not only suggest that B cells play an important role in the induction of CD8 T cells but also that they improve bacterial clearance in DNA vaccine model.

## Background

DNA vaccines consist of the specific gene of interest cloned into a bacterial plasmid that is engineered for optimal expression in eukaryotic cells, thereby leading to protective immune responses. In contrast with subunit vaccines, in DNA vaccination the immunogen is synthesized within the host by cells that have taken up the antigen-encoding DNA. Consequently, DNA vaccines join the advantages of subunit vaccines with the ability to generate antigens endogenously making them accessible to presentation. Moreover, CpG motifs in bacterial DNA can elicit innate immune response via Toll-like receptor 9-dependent pathway interfering with T cell activation [1,2]. One intriguing aspect of DNA vaccination involves the mechanism by which the encoded antigen is processed and presented to the immune system. Studies with bone marrow-chimeric mice have suggested that bone marrow-derived APCs play a key role in the induction of the immune response after DNA vaccination [1]. We previously demonstrated that intramuscular (i.m.) delivery of pcDNA3 encoding *M. leprae *65-kDa heat shock protein (pcDNA3-Hsp65) results in activation of immune response, as well as protection against virulent *M. tuberculosis *challenge [3,4].

Despite the efficacy of DNA vaccines delivered by i.m. injection, little is known about the tissue distribution of plasmid as well as the cells involved in the uptake of pcDNA3-HSP65 *in vivo*. We have previously shown that pcDNA3-HSP65 can be uptaken by B cells *in vivo *[5]. These findings greatly encouraged us to analyze the role of B cell in DNA vaccines.

It was suggested that the uptake of plasmid DNA by B cells after intrasplenic injection also contributes to endogenous synthesis of antigen and presentation [6]. B cells may play a key role in T-cell-mediated immunity either acting as antigen-presenting cells or as antigenic reservoirs, which result in amplification of the immune response. In protein-based vaccines, B cells showed extra and essential antigen presentation capacity over and above that provided by dendritic cells. Furthermore, BCR-mediated uptake may make them efficient APCs even at low Ag doses [7,8]. It was shown that antigen-specific B cells are the main APC responsible for the clonal expansion of antigen specific CD4 T cells in the lymph nodes after protein immunization [8]. On the other hand, B cells can activate CD8 T cells specifically but require CD4 T help. Interestingly, CD8 T cell activation can result either from the presentation of the cytotoxic (CTL) and helper T lymphocytes epitopes by the same antigen presenting B lymphocyte or from a sequential presentation of the two epitopes by two different antigen-presenting B lymphocytes [9].

It has been shown that priming with a low dose of B cells transgenic for the dominant epitope (NP366-374) of the influenza virus nucleoprotein is highly efficient in inducing protective memory CTL response and the protection from disease is mediated by central memory CD8 T cells [10]. Despite the differences between these approaches and DNA vaccination they demonstrated the importance of B cells in inducing memory CD8 T cells.

In the present study the role of B lymphocytes as antigen presenting cells and also in the generation of memory T cells was evaluated *in vivo *after plasmid DNA internalization. The results obtained herein open perspectives to the development of DNA vaccine strategies and highlight the importance of B cells in this model.

## Methods

### Mice and immunization protocol

In this study we used 6- to 8-week-old mice of the following strains: C57BL/6 wild-type (WT) and B cell deficient (BKO; μ chain-/-) mice. The mice were bred and maintained under specific pathogen-free conditions in the animal house of the Medical School of Ribeirão Preto, University of São Paulo, Ribeirão Preto, São Paulo, Brazil. Gene-deficient mice were obtained from the Jackson laboratories (Bar Harbor, ME). All experiments were performed in accordance with the guidelines of the Animal Care Committee (CETEA) of the University, protocol number 172/2005. pcDNA3-Hsp65 and empty plasmid were purified on an endofree QIAGEN plasmid column, as previously described [4]. Mice were injected into quadriceps muscle with 100 μg/mouse of naked plasmid DNA in 25% PBS-sucrose (100 μl total volume; 50 μl each one). The mice were immunized three times in fifteen days interval and euthanized on days 15 and 30 after the last immunization.

### T lymphocytes proliferation assays

The spleens of immunized mice were aseptically collected and cultured in complete RPMI medium [RPMI 1640 (Invitrogen) containing 2 mM L-glutamine, 50 mM 2-mercaptoethanol, 100 units/mL penicillin, 100 mg/mL streptomycin (Sigma-Aldrich Co.), and 10% heat-inactivated fetal calf serum (FCS) (Invitrogen)], in the presence of peritoneal macrophages and 50 μg of recombinant Hsp65 for three days. The T lymphocytes were sorted using anti-CD8 or anti-CD4 antibodies linked to magnetic beads (MACS^® ^MicroBeads System - Miltenyi Biotec). The B lymphocytes were obtained by negative selection (> 90% CD19+) using anti-CD43 antibody linked to magnetic beads (MACS). They were electroporated 300 V, 150 μF and 100 Ω in the GenePulser Xcell™ (Bio-Rad). It was used 10 μg of pcDNA3-Hsp65 or empty vector to 1 × 10^6 ^cells (5 × 10^6^/mL) in Opti-MEM (Invitrogen). The cells were cultured in 96 wells culture cluster, round bottom, 1 × 10^5 ^per well, in complete RPMI medium. After three days, 1 × 10^6 ^CFSE-stained T CD4 or T CD8 lymphocytes were added and cultured for more three days. T cells proliferation was measured by the dilution of intracellular CFSE staining detected by flow cytometry (FACSort™ - BD Bioscience). As positive control some T cells were treated with concanavalin A (4 μg/mL).

### Real-time PCR

After electroporation, the B cells were cultured in 24 wells culture cluster (2 × 10^5 ^per well) for three days. The T lymphocytes (obtained as previously described) were added for additional three days. Then, the cells were collected in 1 mL of TriZol^® ^reagent (Invitrogen) and the RNA was extracted according to the manufacturer protocol. The total RNA was treated with DNAseI amplification grade (Invitrogen) and cDNA synthesis was made using SuperScriptII (Invitrogen) according to manufacturer's instructions. In each real-time reaction was used 200 ng of cDNA (GeneQuant II- Amershan-Pharmacia) and 0,1 μg/μL of each primer (sense and anti-sense). It was used the Platinum^® ^SYBR^® ^Green qPCR SuperMix-UDG (Invitrogen) and the reactions were performed in Rotor-gene (Corbett Life Science, Mortlake, NSW, Australia). The Rotor-gene 6000 software was used to calculate the relative expression of each gene in APCs samples trasnfected with pcDNA3-HSP65 or empty vector compared with the mock electroporated cells. The beta-actin gene was used as integrity samples control and primers sequences were: β-actina sense AGC TGC GTT TTA CAC CCT TT; β-actina anti-sense AAG CCA TGC CAA TGT TGT CT; GATA-3 sense AGG AGT CTC CAA GTG TGC GAA; GATA-3 anti-sense TTG GAA TGC AGA CAC CAC CT; T-bet sense CCC CTG TCC AGT CAG TAA CTT; T-bet anti-sense CTT CTC TGT TTG GCT GGC T; Foxp3 sense ACA ACC TGA GCC TGC ACA AGT; Foxp3 anti-sense GCC CAC CTT TTC TTG GTT TTG.

### Adoptive transfer assays

B lymphocytes were electroporated, as described above and 1 × 10^6 ^cells were injected into the retro-orbital plexus of WT and BKO mice. After seven days, the mice were euthanized for analysis of CD44 and CD62L memory markers on spleen T cells, by flow cytometry in FACSort™(BD Bioscience). The antibodies used were all purchased from BDPharMingen and the analysis was made using the CellQuest^® ^software (BD Biosciences). Another group of mice were challenged with 1 × 10^5 ^virulent *M. tuberculosis *strain H37Rv (ATCC 27294) by intranasal route on day seven post-immunization. Thirty days after the infection, the mice were euthanized, the lungs were collected and the colony-forming units (CFU) of *M. tuberculosis *were determined by serial 10-fold dilution of lung homogenate on Middlebrook 7H11 agar enriched with 10% of heat-inactivated FCS. CFU Petri dishes were incubated at 37°C for 4 weeks and the number of colonies was visually counted. Results are expressed as mean log_10 _values per gram of tissue.

### Statistical Analysis

Statistical determinations of the difference between means of experimental groups were performed using one-way analysis of variance (ANOVA) followed by Tukey post test. Differences that provided *P *value less than 0.05 were considered to be statistically significant.

## Results and Discussion

**Figure 1:**
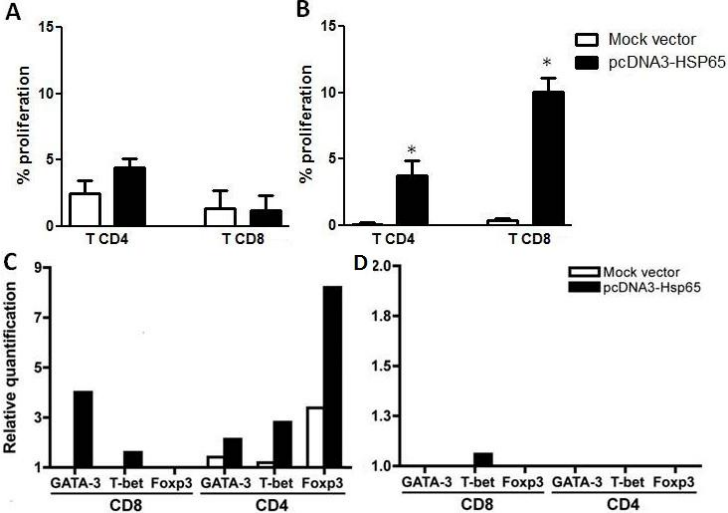
**Proliferation of CD4 and CD8 T lymphocytes obtained after pcDNA3-HSP65 immunization**. (A-B) C57BL/6 wild-type (WT) mice were immunized three times with 100 μg of naked pcDNA3 encoding *M. leprae *65-kDa heat shock protein (pcDNA3-HSP65)/mouse in fifteen days interval. Fifteen (A) or thirty days (B) after the last immunization, the spleens were cultured in the presence of peritoneal macrophages and 50 μg of recombinant Hsp5 for three days to re-stimulate Hsp65 specific cells. CD8 and CD4 T cells were sorted and incubated for three days with different antigen presenting cells that had been previously electroporated with pcDNA3, pcDNA3-Hsp65 or a mock control (medium). The proliferation was measured by CFSE staining dilution by flow cytometry and the mock control values were subtracted from the mock vector and pcDNA3-Hsp65 ones. Assays were performed in triplicate and the results represent the mean ± SD of at least two independent experiments. *P < 0,05 versus other stimuli (One-way ANOVA with Tukey's post-test). (C-D) Gene profile of GATA3, T-bet and Foxp3 from total RNA of both population were evaluated by real-time PCR 15 (C) and 30 (D) days after the last dose of the vaccine and incubated for three days with B cells as previously described. The level of expression was calculated using the mock cell as an internal control. The data is representative of 2 independent experiments.

Plasmid DNA has been delivered *in vivo *to express the encoded gene for several years since 1990. Nevertheless, the mechanisms of immune system activation by DNA vaccines are not complete elucidated [11]. To evaluate whether transfected B cells are able to induce both T lymphocytes proliferation and cytokine production, Hsp65-specific CD4 and CD8 T cells were induced through i.m. immunization of C57BL/6 mice with three doses of pcDNA3-Hsp65 at a 2-week interval. First, we analyzed the proliferation of spleen-sorted CD4 and CD8 T cells, which were obtained on day 15 after the last immunization and stimulated with recombinant Hsp65 for three days. Afterward, these T cells were co-cultured with pcDNA3-Hsp65-electroporated B cells, for three days. By this time point CD4 or CD8 T cells proliferation was not observed (Figure 1A), possibly due to low number of Hsp65-specific T cells. On the other hand, when transfected B cells were co-cultured with spleen-sorted CD4 and CD8 T cells obtained on day 30 after the last immunization, in the same conditions, they were able to induce CD4 and CD8 T cells proliferation, which was significantly higher than that from mock vector-electroporated cells (Figure 1B). This result suggests that pcDNA3-Hsp65-electroporated B lymphocytes can induce T cells activation in our vaccine model.

Since transfected B cells were effective in inducing CD4 and CD8 T cells activation, we asked whether they could modulate the expression of the following T cell transcription factors: T-bet, GATA3 and Foxp3, which are master regulators of Th1, Th2 and regulatory T cell development respectively. To this end, T-bet, GATA3 and Foxp3 expression were examined by real-time RT-PCR after *in vitro *stimulation. Analysis of T cells obtained on day 15 after the last immunization and cultured with transfected APC showed that B cells were able to induce GATA-3 and T-bet expression in CD8 T cells, while the three transcription factors were expressed in CD4 T cells (Figure 1C). On the contrary, when T cells obtained on day 30 after the last immunization were cultured with B cells, T-bet expression occurred only in CD8 T populations (Figure 1D). This remaining T-bet expression can drive the interferon-γ production. These findings indicate that transfected B cells can modulate CD4 and CD8 T cells activation profile and additionally play an important role in the development of CD8 T cells immune response.

**Figure 2:**
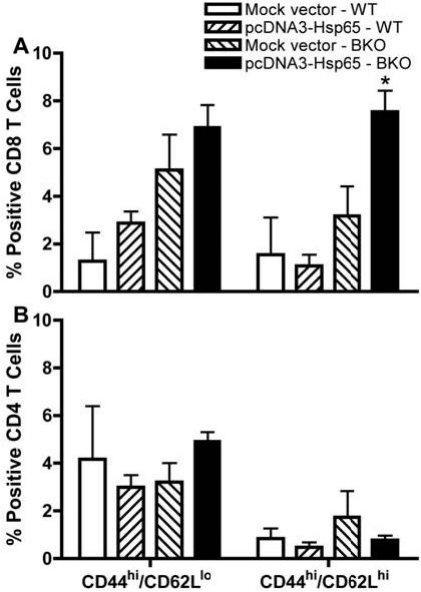
**Phenotypical analysis of memory cells in the spleen of reconstituted BKO mice**. 1 × 10^6 ^electroporated B lymphocytes (pcDNA3-Hsp65 or mock vector) were injected by endovenous route (orbital plexus) in WT and B cell deficient mice. After seven days, the mice were euthanized for analysis of CD44 and CD62L on CD8 (A) and CD4 (B) T cells, by flow cytometry. Assays were performed in triplicate and the results represent the mean ± SD of at least two independent experiments. *P < 0,05 versus wild-type mice (One-way ANOVA with Tukey's post-test).

**Figure 3:**
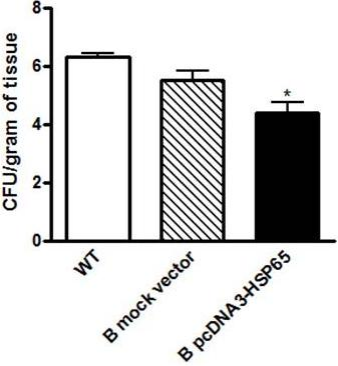
**Colony forming units (CFU) of *M. tuberculosis *in the lung of infected mice**. The BKO mice which received electroporated B cells and control wild-type mice, were inoculated with 1x10^5 ^M. tuberculosis. Thirty days post infection, the CFU in the lung homogenates were analyzed. Assays were performed in triplicate and the results represent the mean ± SD of at least two independent experiments. *P < 0,05 versus wild-type mice (One-way ANOVA with Tukey's post-test).

In order to evaluate the true value of B cells in antigen presentation, this APC electroporated with both pcDNA3-Hsp65 and empty vector was adoptively transferred to B cell-deficient mice (BKO) and on day 7 after this procedure the phenotype of CD4 and CD8 T cell populations from spleen were analyzed by flow cytometry. As shown in Figure 2A, pcDNA3-Hsp65-tranfected B cells adoptively transferred into BKO mice were able to induce a significant increase in the percentage of central memory (CM) CD8^+ ^CD44^hi ^CD62L^hi ^T cell subsets [12] when compared with WT mice that received the same treatment. On the other hand, there was no significant difference in the percentage of CM CD4^+ ^CD44^hi ^CD62L^hi ^T cell subsets (Figure 2B). These *in vivo *assays not only show that B cells were able to act as APC but also that they had capacity to activate CD8 T lymphocytes and promote the generation of memory CD8 T cells. These *in vivo *results are in agreement with the above findings where B cells presented an important role mainly in the development of CD8 T cells immune response. Our results are consistent with data of Castiglioni et al. [10] where transgenic B cells were used as APC vaccine and were highly efficient in inducing anti-virus protective immunity mediated by central memory CD8 T cells. On the basis of these results we next evaluated the role of transfected B cells adoptively transferred in experimental tuberculosis. The role of B cells in the protection against experimental tuberculosis has been reported as a controversial issue [13,14] which might be due to the several animal models and the variety of both routes and strains used. CFU recovery from the lungs, an important parameter to evaluate the course of experimental tuberculosis showed that BKO mice that were injected with pcDNA3-Hsp65-electroporated B cells presented a significant reduction in the number of CFU when compared with WT mice. Curiously, there was no difference in the number of CFU recovered from the lungs of BKO mice immunized with both the vector-transfected cells and pcDNA3-Hsp65 B cells (Figure 3). These results may be more attributed to a genetic pattern than to protein levels, showing the importance of the B cell mediated immune response in our model. Indeed, it has been shown that B cells are required to prime memory T cell responses, placing the B and T cell relationship in relevant context to vaccine biology [15].

Taken together, these results demonstrated that during the activation of the immune system B cells can modulate the response, mainly by inducing CD8 T lymphocytes. This modulation can be responsible for inducing protection. Additionally, that is the first time that an *in vivo *DNA-vaccine model showed the importance of B cells in CD8 T cell activation.

## Conclusion

The immune response against DNA-Hsp65 showed a complex network of APC types and nuclear cytokine factors. We are the first to demonstrate the participation of pcDNA3-Hsp65-electroporated B lymphocytes as antigen presenting cells able to induce CD8 T cells activation. Overall, these data open perspectives to study B cells and how they can be harnessed to optimize the DNA vaccine strategies and consequently the immunity against M. tuberculosis.

## Competing interests

The authors declare that they have no competing interests.

## Authors' contributions

LPA performed the research, analyzed the data and wrote the paper. APFT processed samples and performed the research. JCCL provided help in the experiments of real-time PCR. ICF, TM and RLLS processed samples. CDR and AFG provided help in the cell culture experiments. CLS provided vital reagents and APC commented on the paper. AMCC designed and supervised the research, analyzed the data and wrote the paper. All authors read and approved the final manuscript.
